# 3D-Printable Chontaduro (*Bactris gasipaes*) Gel Inks: Influence of Encapsulated *Lactiplantibacillus plantarum* on Rheological, Textural, and Sensory Properties

**DOI:** 10.3390/gels12050390

**Published:** 2026-05-01

**Authors:** Annamaria Filomena-Ambrosio, Luz-Indira Sotelo-Díaz, Yeison-Fernando Barrios-Rodríguez, Diana Vicente-Jurado, Stephania Aragón-Rojas, María Ximena Quintanilla-Carvajal, Marta Igual, Javier Martínez-Monzó, Purificación García-Segovia

**Affiliations:** 1 PhD Programme in Food Science, Technology and Management, Universitat Politècnica de València, Camí de Vera s/n, 46022 Valencia, Spain or anna.filomena@unisabana.edu.co (A.F.-A.); yfbarrod@upv.es (Y.-F.B.-R.); diaviju@doctor.upv.es (D.V.-J.); 2 Research Group in Alimentación, Gestión de Procesos y Servicio, International School of Gastronomy Program, Economic and Administrative Sciences, Universidad de La Sabana, Campus del Puente del Común Km. 7, Autopista Norte de Bogotá, Chía 250001, Colombia; indira.sotelo@unisabana.edu.co (L.-I.S.-D.);; 3 i-Food Research Group, Instituto Universitario de Ingeniería de Alimentos-FoodUPV, Universitat Politècnica de València, Camí de Vera s/n, 46022 Valencia, Spain; xmartine@tal.upv.es (J.M.-M.); pugarse@tal.upv.es (P.G.-S.); 4 Research Group in Agroindustrial Process, Engineering Faculty, Universidad de La Sabana, Campus del Puente del Común Km. 7, Autopista Norte de Bogotá, Chía 250001, Colombia; maria.quintanilla1@unisabana.edu.co

**Keywords:** *Lactiplantibacillus plantarum*, rheological, sensorial analysis, chontaduro gel

## Abstract

This study evaluated the feasibility of developing 3D-printable chontaduro (*Bactris gasipaes*) gel inks. Freeze-dried chontaduro pulp and encapsulated *Lactiplantibacillus plantarum* were used. Two formulations were analysed: a control (ChC) and a probiotic ink (ChLp) containing 10% (*w*/*w*) microencapsulated cells in a maltodextrin–whey protein carrier. Both were baked at 140 °C under zero humidity and evaluated for water activity, colour, texture, and sensory properties. Rheological analysis showed shear-thinning behaviour for both inks. Notably, ChLp had higher storage (G′) and loss (G″) modulus, which may indicate structural reinforcement by the carrier. Furthermore, FTIR suggested enhanced protein–polysaccharide interactions and ionic cross-linking. Both inks were found to be extrudable; however, ChLp showed a 4.1% reduction in printed height. Baking reduced water activity (aw < 0.88) and caused Maillard browning, which was more pronounced in ChLp. With respect to microbial viability, *Ltp. Plantarum* viability (~7.1 log CFU/g) was maintained after extrusion but lost after baking. Sensory evaluation indicated formulation-dependent differences in colour (greater yellowness) and texture (reduced adhesiveness, increased hardness) for ChLp. Overall, these findings showed chontaduro gel as a viable matrix for 3D food printing, with the encapsulated carrier altering physicochemical and sensory descriptors.

## 1. Introduction

Three-dimensional (3D) food printing is a disruptive technology that enables both nutritional and structural customisation. Among the various techniques, extrusion-based printing stands out for its versatility in processing food inks rich in fibre, proteins, and starches, thereby facilitating the creation of complex designs using raw materials that are often underutilised [1,2,3]. This technology also enables the production of personalised foods tailored to individual nutritional requirements, including adapted supplements, specialised diets for patients, or the encapsulation of microorganisms [4,5,6]. One of the main challenges in 3D food printing is preserving nutritional quality, as processes such as high-temperature and high-pressure extrusion can induce biochemical reactions including protein denaturation, starch gelatinisation, and possible vitamin degradation, thereby affecting the bioavailability of micronutrients [7,8].

Three-dimensional food printing can also integrate novel ingredients into conventional products, replicating the appearance of nutritious but less widely accepted food options to enhance their consumer acceptability and consumption [9,10]. Likewise, it can improve the digestibility of macronutrients, such as proteins [11,12,13]. This approach addresses challenges within the food supply chain by simplifying production, adding value to by-products, and expanding the diversity of raw materials, including tropical fruits with high functional potential [14,15] such as chontaduro (*Bactris gasipaes*).

Chontaduro (*Bactris gasipaes*) is the fruit of a palm native to the tropical regions of the Americas, belonging to the Arecaceae family, the same group that includes açaí and coconut. Depending on the species, its colour varies from deep red to orange, yellow, or green [16,17]. The pulp of chontaduro is characterised by a high content of bioactive compounds, particularly β-carotenes [18], which are associated with positive health effects. Innovation lies in revalorising chontaduro (*Bactris gasipaes*) as a raw material for 3D printing by leveraging its fibre and carotenoid content [19] as potential functional foods. The incorporation of microorganisms into food matrices for 3D printing represents a promising design strategy, as their biological activity may act as a structuring agent influencing rheological properties [20] and sensory attributes related to flavour and texture [21].

The aim of this study was to evaluate the technical and functional feasibility of 3D-printable doughs based on freeze-dried chontaduro (*Bactris gasipaes*) pulp, formulated with encapsulated *Lactiplantibacillus plantarum*, as a case study for the development of functional foods through 3D printing.

## 2. Results and Discussion

### 2.1. Influence of Lactiplantibacillus plantarum Incorporation on Chontaduro Gel Ink

#### 2.1.1. Rheological Characteristics

**Figure 1:**
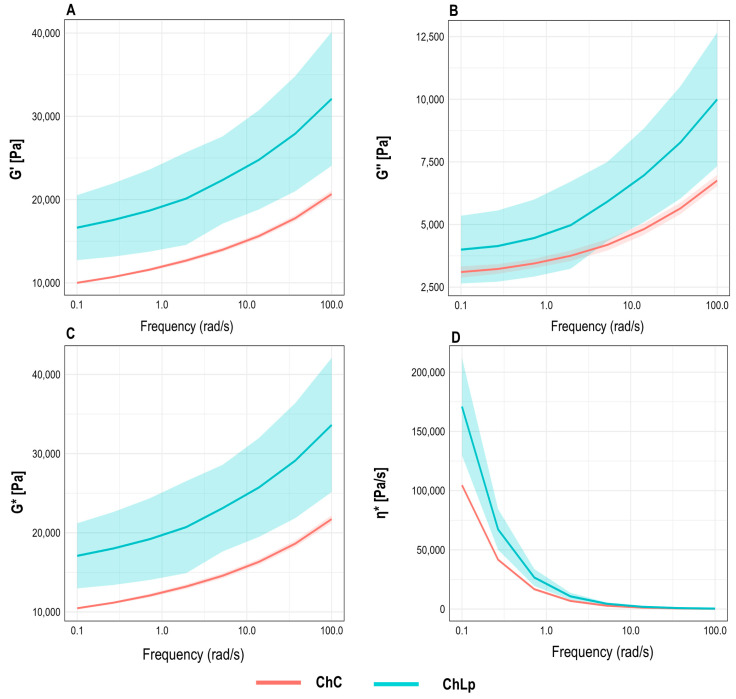
Comparison of viscoelastic parameters: storage modulus (**A**), loss modulus (**B**), complex modulus (**C**), and complex viscosity (**D**) of ink control chontaduro gel (ChC) and in chontaduro gel with *Lactiplantibacillus plantarum* (ChLp) with respective 95% confidence intervals (*n* = 3).

The printing ink was characterised to assess its rheological behaviour, as viscoelastic properties are crucial for printability and structural stability in 3D food printing. Frequency sweep profiles revealed the storage modulus over the entire frequency range [22]. Both formulations showed a progressive increase in storage modulus (G′) (Figure 1A) with frequency, indicating a stronger structural network against rapid deformation.

The ChLp formulation achieved significantly higher values than the control, confirmed by the absence of overlap in the 95% confidence intervals (95% CIs) across most of the range. These results suggest that encapsulated microorganisms promoted a more rigid and elastic network via physical interactions between the maltodextrin and the protein–polysaccharide matrix. The loss modulus (G″) (Figure 1B) also increased with frequency, but stayed lower than G′. ChLp’s magnitude exceeded that of the control, with 95% CIs consistently separating at intermediate and high frequencies. Overall, these results indicate greater viscoelastic rigidity in the presence of the *Lactiplantibacillus plantarum* carrier. Higher complex viscosity at low frequencies suggests greater structural resistance at rest, whilst the convergence at high frequencies shows comparable pseudoplastic behaviour for both formulations. The complex modulus (G*) (Figure 1C) followed the same trend, confirming greater overall mechanical strength for ChLp. Wider confidence bands at high frequencies reinforce the robustness of treatment difference. Complex viscosity (η∗) (Figure 1D) for both inks sharply decreased with frequency, reflecting pseudoplastic (shear-thinning) behaviour. This profile benefits 3D printing by facilitating nozzle extrusion under stress while maintaining viscosity to sustain the structure after deposition [23]. *Ltp. plantarum* ink had higher η* values in the low frequency range, suggesting greater resistance to flow at rest [24]. At high frequencies, converged curves indicate that microcapsules induced structural reinforcement which attenuates under rapid dynamic deformations.

These observations indicate that introducing the microorganism alters the viscoelastic response of the food ink. Elevations in G′, G″, and G*, alongside increased viscosity at low frequency, collectively contribute to enhanced structural and printability [22]. The results suggest that the incorporation of this microorganism serves not only as a biological carrier but also as a structural agent within the matrix, providing notable technological advantages in additive food manufacturing.

#### 2.1.2. Spectral Characteristics

**Figure 2:**
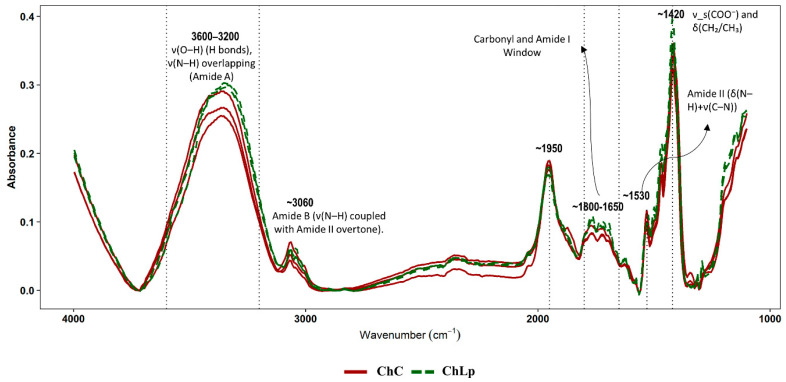
The infrared spectrum of food inks including *Lactiplantibacillus plantarum* (ν is stretching vibration and δ is bending) (control chontaduro gel: ChC; chontaduro gel with *Lactiplantibacillus plantarum*: ChLp).

The evaluation of spectral and colorimetric characteristics provides insights into the molecular composition and visual attributes of the food inks. Fourier transform infrared spectroscopy (FTIR) analysis identified representative absorption bands associated with proteins, polysaccharides, lipids, and water (Figure 2). In particular, the broad envelope at 3600–3200 cm^−1^ was assigned to ν(O–H)/ν(N–H) stretching vibrations [24,25]. The band around 3060 cm^−1^, attributable to protein Amide B [26], showed minor differences suggesting modifications in the N–H environment rather than a profound structural transformation. The 1800–1650 cm^−1^ window is dominated by Amide I [24], with strong overlap from δ(H–O–H) at ~1640 cm^−1^ [26]. The subtle variations in shape and intensity may suggest minor modifications in the protein environment rather than a significant conformational transition. Amide II, associated with the wave number ~1530 cm^−1^ [25], also shows fine adjustments that could be compatible with the protein–polysaccharide matrix of the *Ltp. plantarum* formulation, suggesting a more cohesive network at rest with less relaxation. This idea is also supported by the possible ionic cross-linking of carboxylates (~1420), which may partially contribute to the consistently higher G′, G″, and G* values and higher η* at low frequencies observed in this formulation.

#### 2.1.3. Printability

**Figure 3:**
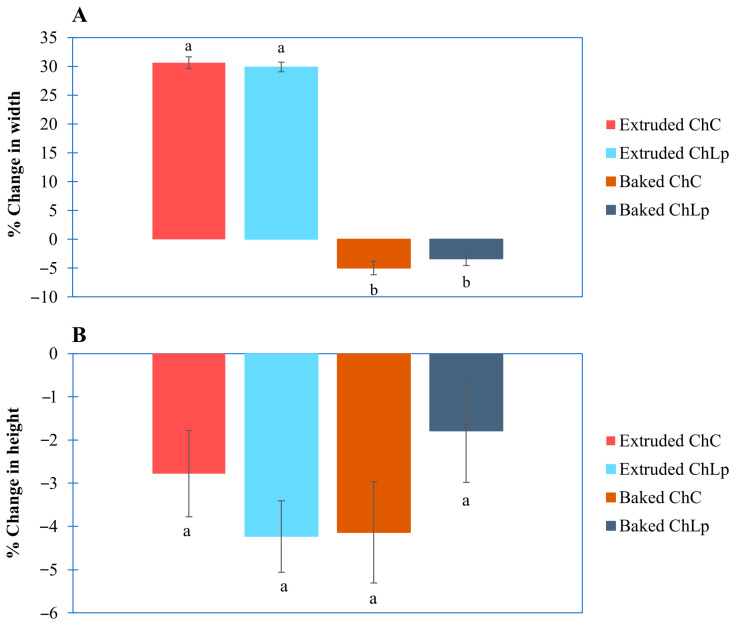
Means and standard error of the percentage changes in the shape of the figures with respect to their theoretical dimensions as a function of width (**A**) and height (**B**). The letters a and b indicate that there are significant differences between the samples based on Tukey’s HSD test (*p* < 0.05) (control chontaduro gel: ChC; chontaduro gel with *Lactiplantibacillus plantarum*: ChLp).

Figure 3 illustrates the dimensional deviations of the printed and baked figures relative to their theoretical dimensions (Section 2.4). For the extruded samples, no statistically significant differences (*p* > 0.05) were found between ChC and ChLp formulations in terms of printability. However, both samples deviated from the intended geometry. The printed height was 2.8 ± 0.6% and 4.1 ± 1.2% lower than expected for ChC and ChLp, respectively, while the width increased by approximately 30% (30.6 ± 0.3% and 29.9 ± 0.4%).

These results indicate that although material deposition was achieved, the formulations did not adequately support the designed structure, leading to lateral expansion, height loss, and partial structural collapse.

Zheng et al. [27] and Liu et al. [28] have reported a correlation between the rheological properties and the structure fidelity of printed constructs, where a higher storage modulus (G′) and hardness promote resistance to deformation. In this study, despite exhibiting a higher G′, the ChLp samples experienced greater height loss than the control, suggesting that an increased elastic modulus alone is insufficient to ensure macroscopic stability. This behaviour may result from the combined effects of extrusion pressure, gravitational forces, and possible over-extrusion phenomena, which favour lateral spreading of the printed layers [29].

When these results are compared with those for the baked samples, significant differences in width (*p* < 0.05) are observed between the extruded and baked samples, regardless of *Ltp. plantarum* presence. This effect is attributed to shrinkage induced during baking (−5.0% ± 0.6% and −3.3% ± 1.1% for the ChC and ChLp samples, respectively), resulting from water loss during thermal treatment [30].

### 2.2. Water Activity (a_w_)

The statistical analysis, as determined by Tukey’s multiple comparisons test, shows a significant effect of both processing and *Lactiplantibacillus plantarum* incorporation on the a_w_ (Table 1). The most insightful factor was the baking process, which resulted in a highly significant reduction (*p* < 0.0001) in a_w_ for both ChC and ChLp when baked, with mean differences of 0.09805 and 0.1111, respectively, compared to the chontaduro gel ink. Baking involves high-temperature heating which causes water evaporation. As free water is removed from the product matrix, the a_w_ decreases significantly. This is a well-documented direct effect of baking and drying processes [31]. Reducing the a_w_ to below 0.88 is a crucial preservation step, as it inhibits the growth of most bacteria, including many pathogens and spoilage microorganisms. This enhances the product’s shelf-life and safety, which is particularly important for a high-moisture ingredient like chontaduro.

On the other hand, *Lactiplantibacillus plantarum* was incorporated into chontaduro-based products using spray-drying encapsulation technologies with hydrocolloids, such as maltodextrin and sweet whey proteins, as carrier materials. The incorporation resulted in a lower a_w_ across all product stages. This effect was statistically significant in the ChLp ink (mean diff. = 0.02480, *p* < 0.0001) and was most pronounced in the baked dough (mean diff. = 0.03788, *p* < 0.0001). The decrease in a_w_ can be attributed to the higher solids content introduced by the encapsulating matrix and the water-binding capacity of its components. Maltodextrin, lactose, and whey proteins are hydrophilic solutes that interact with water molecules, lowering the vapour pressure and thus decreasing the measured a_w_ in the system [31,32]. This formulation-related reduction in a_w_ complements the effect of baking and contributes to the overall stability of the product.

### 2.3. Colour

The colour stability of plant-based matrices rich in β-carotenes, such as chontaduro, is a critical quality indicator. It reflects the structural integrity of these bioactive pigments during processing [33]. The incorporation of functional ingredients, such as *Lactiplantibacillus plantarum*, and the use of technological processes, including 3D printing, extrusion, and baking, can induce significant physicochemical changes (Table 2).

The increase in b* and C* indicates a more intense, saturated yellow colour in the ink and extruded dough ChLp. This can be attributed to the initial interaction between the encapsulating matrix and the native carotenoids from chontaduro. The sweet whey protein and maltodextrin matrix could create a more homogeneous dispersion of the lipid-soluble carotenoids within the ink’s aqueous phase. This system may form a protective colloidal structure that enhances light scattering and the perception of yellowness. Similar interactions have been reported in plant-based systems. There, the addition of proteins and polysaccharides can modulate the optical properties and improve carotenoid stability by preventing their aggregation [34]. The slight but significant increase in L* supports the hypothesis of a more light-scattering, homogeneous microstructure induced by the bacterial carrier [35].

For the control sample ChC, the extrusion process did not induce statistically significant changes in colour parameters compared to non-extruded ink, except for a*, where these differences were minimal. This remarkable stability suggests that the native chontaduro matrix, rich in fibres and other biopolymers, provided a protective environment for the β-carotenes during the extrusion process, which subjected them to shear stress [16].

The extruded ChLp dough remained significantly lighter (higher L*, *p* < 0.0001). It also exhibited higher yellowness (b*, *p* < 0.0001) and chroma (C*, *p* < 0.0001) compared to the extruded control (ChC). Research on the 3D printing of functional foods has shown that encapsulating agents, such as whey protein and maltodextrin, can protect bioactive compounds and microorganisms from the mechanical stress of extrusion. This ensures their stability and functionality in the printed construct [35]. The analysis of colour differences showed minimal variations (Δ*E* = 3 ± 2), falling within the range of human eye detection. Studies by other researchers [36] also resulted in changes in the colouration of the control samples after the addition of *Ltp. plantarum*. Concurrently, authors such as Gutiérrez-Álzate et al. [37] have examined the impact of encapsulation on the colour transformation of specimens, as it can influence the release of the microorganism.

Baking, a high-temperature, low-moisture process, induced statistically significant changes in the colour of all samples (*p* < 0.0001 for most parameters). However, the effect was drastically different between the ChC and ChLp doughs. In the ChC dough, baking caused a significant decrease in L* and an increase in a*, showing darkening and a shift towards a more reddish hue. These are classic signs of non-enzymatic browning reactions, such as the Maillard reaction and caramelisation [35]. Two non-exclusive mechanisms could explain this phenomenon. The first involves the oxidative thermal degradation of major carotenoids in chontaduro, such as β-carotene and lycopene. This leads to molecular cleavage, the forming of colourless epoxides and volatile, low-molecular-weight compounds. Such products directly cause the observed loss of chroma (maintained a* value but decreased b* value) and the darkening (decreased L*) [16,38]. Secondly, the use of sweet whey protein as a carrier for bacteria probably contributed to the pronounced darkening (L* = 44.5 ± 0.6, *p* < 0.0001). This finding aligns with previous studies that show that whey protein concentrates (WPCs) promote non-enzymatic browning during baking. Pérez et al. [39] attributed a similar darkening in baked goods to Maillard reactions between residual lactose in WPCs and the free amino groups, particularly those from lysine, inherent to whey proteins. The lysine content in whey protein contributes to the dark colour, as it is the main reactive amino group that reacts with reducing sugars. Pérez et al. [39] supported this hypothesis.

### 2.4. Texture Profile

Table 3 shows the textural profile of 3D-printed foods, a critical quality attribute that is directly influenced by formulation and post-processing. The most striking observation is the transformative effect of baking, which induced statistically significant increases (*p* < 0.0001) in all textural parameters for both formulations compared to their extruded dough. However, the key finding lies in the differential impact of incorporating the bacteria. In the extruded dough, the addition of the microorganism carrier did not result in any statistically significant alterations to hardness, cohesiveness, springiness, gumminess, or chewiness. This is a crucial technological advantage, indicating that the inclusion of *Ltp. plantarum* (10% *w*/*w*) does not detrimentally affect the extrudability or the initial texture of the 3D-printed construct. This stability can be attributed to the similar rheological-modifying properties of the maltodextrin and sweet whey-based *Ltp. plantarum* carrier compared to the original formulation components. The high shear forces during extrusion likely homogenise the matrix sufficiently to mask any minor structural differences, a phenomenon observed in other protein–polysaccharide composite gels used for 3D printing [28].

The texture profile changes after baking. Both formulations underwent significant hardening. The ChLp dough became significantly harder, gummier, and chewier than the ChC baked dough (*p* < 0.0001 for hardness and gumminess; *p* < 0.01 for chewiness). This pronounced textural strengthening can be linked to the specific composition of the *Ltp. plantarum* carrier and its interaction during thermal processing. The carrier, a maltodextrin and sweet whey blend in a 0.6:0.4 ratio, introduced a high concentration of two key reactive components: dairy sugars (lactose) from the sweet whey and proteins (whey proteins).

### 2.5. Characterization of Viability Lactiplantibacillus plantarum in Chontaduro Doughs

The viability of *Ltp. plantarum* is a critical parameter for the development of functional foods, as 10^6^ CFU/g of live cells must reach the intestines to confer health benefits Table 4 shows the evolution of the *Ltp. plantarum* strain incorporated into a chontaduro-based matrix, subjected to the physical stress of extrusion and the biochemical challenges of the human gastrointestinal tract (GIT).

The initial viability of *Ltp. plantarum* in the chontaduro gel ink was 7.01 ± 0.18 log CFU/g, showing a high baseline (Table 4). No significant reduction occurred after extrusion. The bacteria in the extruded dough maintained a count of 7.12 ± 0.11 log CFU/g. This retention of viability is noteworthy. The extrusion process during 3D printing exposes bacterial cells to mechanical shear forces and potential thermal stress. The lack of a significant decrease suggests a protective effect of the chontaduro matrix. This protection may come from the dense, fibrous matrix structure, which can act as a physical barrier. The barrier could reduce the impact of shear stress on bacterial cell walls [40]. Yoha et al. [41] also reported that *Ltp. plantarum* viability could be retained after 3D printing.

The trajectory of cell survival through the simulated GIT reveals the strain’s susceptibility to specific digestive hurdles. In both the ink and extruded dough, the transition from the pre-digestion stage to the oral phase resulted in a minor, statistically insignificant decline in viability (7.01 to 6.84 Log CFU/g for the ink, and 7.12 to 6.95 Log CFU/g). This indicates that the short exposure to salivary enzymes and the neutral pH of the oral phase posed little threat to the *Ltp. plantarum* cells.

The statistically significant reduction in viability occurred during the gastric phase for both formulations (*p* < 0.05). ChLp gel viability dropped to 5.56 ± 0.83 log CFU/g, while the ChLp extruded dough experienced a more severe decline to 4.74 ± 0.20 log CFU/g. The gastric phase primarily challenges bacteria through low pH (typically 2.0–3.0) and the presence of pepsin. The significant drop (exceeding 1 log reduction in the extruded dough) is characteristic of acid stress [41]. The physical stress of extrusion may have sub-lethally injured a portion of the bacterial population, compromising the integrity of the cell membrane or stress response systems, thereby rendering them more vulnerable to subsequent acid stress in the stomach. This phenomenon of pre-stress sensitization is a critical consideration in *Lt. plantarum* food design; for example, spray-dried microorganisms demonstrated that processing-induced sub-lethal injury can significantly reduce a strain’s gastric tolerance, as the energy required for cellular repair competes with the resources needed for acid resistance mechanisms [42].

In baked doughs, thermal treatment led to the complete inactivation of *Lactiplantibacillus plantarum* at the cellular level. Consequently, the microorganism was not viable prior to the digestion process, and therefore, the simulated gastrointestinal digestion could not be performed on these samples. Nevertheless, as previously discussed, the bacterium’s presence had a significant impact on the physicochemical characteristics of the samples. Specifically, the presence of lactic acid bacteria, such as *Ltp. plantarum*, and its components, such as exopolysaccharides, can contribute to improvements in the rheological and textural properties of the samples [43]. Furthermore, Mishra et al. [44] reported that the changes induced by cell components or death batteries can be perceptible to consumers and improve both the sensory and physicochemical attributes of foods. For these reasons, a sensory evaluation was conducted to determine whether *Ltp. plantarum* could enhance the sensory attributes of the 3D-printed chontaduro gel samples.

### 2.6. Sensorial Analysis

**Figure 4:**
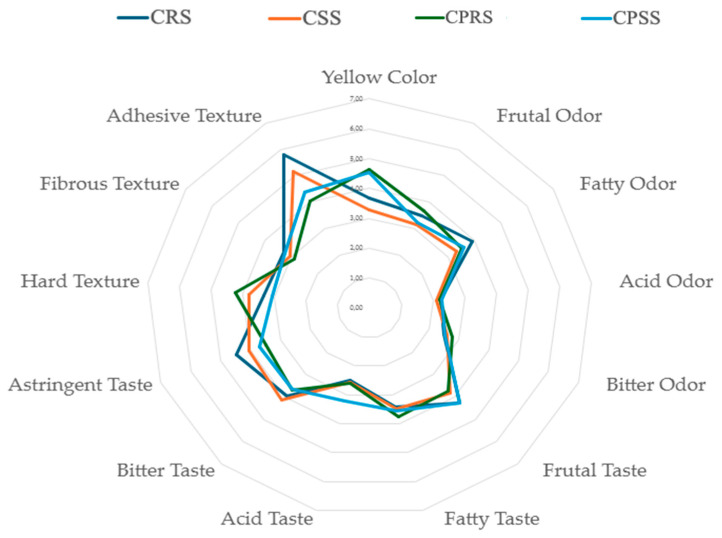
Sensory evaluation of multiple attributes using a radial graph of baked dough with and without the *Lactiplantibacillus plantarum* (blue line is chontaduro dough with a round shape (CRS); orange line is chontaduro dough with a square shape (CSS); green line is chontaduro baked dough with *Ltp. plantarum* in a round shape (CPRS); light blue line is chontaduro baked dough with *Ltp. plantarum* in a square shape (CPSS)).

During baking at 140 °C for 35 min under zero humidity, two primary reactions are intensified. First, the Maillard reaction between the lactose and the amino groups from the whey proteins (both from the original formulation and the carrier) and from the chontaduro itself could be accelerated. The composition of melanoidins and cross-linked protein aggregates directly contributes to structural hardening and darkening. These findings align with colour analysis. In merengue cookies with sweet whey protein–maltodextrin conjugates, adding dairy ingredients rich in lactose consistently increases firmness and browning due to advanced Maillard cross-linking [45]. Second, the high concentration of maltodextrin, a low-dextrose equivalent carbohydrate, undergoes extensive glass transition and recrystallization upon baking and subsequent cooling under low humidity. This behaviour occurs because maltodextrin molecules have many hydroxyl groups which can form hydrogen bonds with water molecules, resulting in a dense network that stabilises the structure [46]. Thus, a rigid, continuous glassy matrix embeds the other components and significantly increases mechanical strength, gumminess, and chewiness. The synergistic effect of protein cross-linking and carbohydrate glass formation in the ChLp sample creates a far more robust, rigid network than in the control (ChC), which lacks the high concentration of reactive sugars and maltodextrin. On the other hand, the sensory evaluation of 3D-printed chontaduro-based baked doughs reveals insights into how both formulation (incorporation of *Ltp. plantarum*) and geometry (shape) influence panellists’ perception (Figure 4). Statistical analysis using Šídák’s multiple-comparisons test indicates that most attributes remained unaffected. However, key visual and textural characteristics were significantly altered. Formulation exerted a stronger effect than geometry.

The most pronounced and statistically significant effect was observed in the visual attribute of yellow colour. The chontaduro-based baked product with *Ltp. plantarum,* both round (CPRS) and square (CPSS), was perceived as significantly more yellow than its control counterparts with the same square shape (CSS vs. CPRS, *p* = 0.0020; CSS vs. CPSS, *p* = 0.0081). This carrier is rich in maltodextrin and sweet whey. It likely participated in Maillard reaction pathways during baking, leading to the formation of yellow brown melanoidins [16]. This intensification of colour could be a positive attribute. Visual appeal is a primary driver of consumer acceptance for novel foods [47].

A paramount finding from product descriptive samples showed high taste and flavour attributes. The incorporation of the 10% *w*/*w* microorganism carrier did not introduce detectable off-flavours. The stability of the fruity taste is particularly important, as it is a characteristic flavour of the base fruit. This neutral flavour profile of *Ltp. Plantarum* is essential for the successful development of functional foods. It should not compromise the sensory quality of the final product [48].

The most striking textural changes were observed in terms of adhesiveness. The control samples, regardless of shape, were perceived as significantly more adhesive than *Ltp. plantarum* (CRS vs. CPRS, *p* < 0.0001; CRS vs. CPSS, *p* = 0.0012; CSS vs. CPRS, *p* = 0.0297). The reduction in adhesiveness in doughs containing *Ltp. plantarum* could be directly linked to their altered composition and the resulting microstructure after baking. As discussed in the textural analysis, the doughs with bacterial development have a harder, more rigid structure due to matrix reinforcement from glassy maltodextrin and protein cross-linking [43]. Moreover, it is also a positive sensory improvement because high adhesiveness is often associated with a negative mouthfeel and poor swallowability [48].

For hardness, a significant difference was found only between the two samples with *Ltp. plantarum* depending on the shape, with the round (CPRS) perceived as harder than the square (CPSS) (*p* = 0.0195). This result highlights a non-intuitive interaction between formulation and geometry. While probiotic products generally increased instrumental hardness, the square shape may have influenced fracture mechanics during chewing, leading to perceived softness compared to its round counterpart. This underscores that, in 3D-printed foods, the geometry is not only a visual attribute but an active design parameter that can modulate texture perception, a principle explored in the field of food structure design [49].

## 3. Conclusions

The use of native bioresources such as chontaduro (*Bactris gasipaes*) in 3D food printing represents a technological challenge, as their natural gel characteristics must be tailored and reinforced, for instance through the incorporation of encapsulated microorganisms, to achieve structural stability and functional performance. In this context, this study evaluated chontaduro gel inks formulated with encapsulated *Lactiplantibacillus plantarum* for 3D printing. The inks demonstrated shear-thinning behaviour and viscoelastic properties (G′ > G″), facilitating nozzle extrusion. Nevertheless, printability assessment demonstrated considerable dimensional deviations: printed figures exhibited a width increase of approximately 30% and a height reduction of up to 4.1% compared to theoretical dimensions, independent of *Ltp. Plantarum* presence. These findings indicate limited shape fidelity, characterised by lateral spreading and partial structural collapse after deposition. Therefore, while the chontaduro gels are extrudable, claims regarding post-extrusion shape retention should be made cautiously.

The addition of 10% *w*/*w* *Ltp. Plantarum* improved techno-functional properties while maintaining printability. Baking at 140 °C fully eliminated microbial viability. The carrier significantly reduced perceived adhesiveness and increased instrumental hardness, as confirmed by sensory analysis. The maltodextrin–whey protein carrier acted as a structuring agent, markedly raising G′, G″, and viscosity at low frequencies, thereby enhancing network cohesion. This research combined rheological, textural, and sensory analyses of chontaduro gels, demonstrating that this underexplored matrix holds promise for 3D food printing by exhibiting suitable rheological behaviour and functional adaptability. During baking, the shape has a positive effect on the hardness of both formulations. However, products not formulated with the microorganism are perceived as less yellow and stickier. Future research should focus on optimising the gel network and evaluating post-processing performance to expand the potential of chontaduro-based systems for functional, sustainable food design.

## 4. Materials and Methods

### 4.1. Raw Material Preparation

#### 4.1.1. Obtaining Freeze-Dried Powder from Chontaduro Pulp (Bactris Gasipaes)

Fresh chontaduro (*Bactris gasipaes*) fruits were manually selected from a local market in Chía, Cundinamarca, Colombia, discarding those exhibiting mechanical damage, signs of over-ripeness, or visible microbial contamination. The selected fruits were washed with potable water and disinfected using a 200 ppm sodium hypochlorite solution for 10 min, followed by rinsing with potable water.

The fruits were then cooked at 100 °C for 5 min using a Rational oven (Landsberg am Lech, Germany). After cooking, the fruits were manually peeled and mechanically de-pulped. The resulting pulp was homogenized in a Thermomix (Vorwerk SE & Co. KG, Wuppertal, Germany) at 3100 rpm (equivalent to speed setting 6) for 5 min at 70 °C. This temperature was selected based on the starch gelatinization range reported by Neto et al. [48] for chontaduro flours.

The homogenized pulp was packed in Ziploc bags and dried in a Labconco freeze-dryer (model FreeZone 12 L, Kansas, MO, USA). The pulp was first frozen at −40 °C with a cooling rate of 0.1 °C/min for 24 h, followed by primary drying at −15 °C and 0.03 mBar for 10 h. Secondary drying was performed at 20 °C and 0.03 mBar for 10 h. Then, the dried product was ground and further homogenized in the Thermomix at maximum speed for 1 min to obtain a fine chontaduro powder.

#### 4.1.2. Preparation of *Lactiplantibacillus plantarum* Strain

The *Lactiplantibacillus plantarum* strain was provided by the laboratory collection of the Universidad de La Sabana. *Ltp. plantarum* was cultivated in a 1 L bioreactor at 37 °C with agitation at 100 rpm for 10 h. The sterile culture medium consisted of 8% (*w*/*v*) sweet whey (Colanta, Colombia) and 0.22% (*w*/*v*) yeast extract (Oxoid Ltd., Basingstoke, UK). The inoculum was added at a 10% (*v*/*v*) concentration. After 10 h of incubation, the culture was mixed with a powdered carrier composed of maltodextrin (Shandong WNN Industrial, Shandong, China) and sweet whey (Colanta, Colombia) in a 0.6:0.4 ratio. Once a homogeneous mixture was obtained, it was subjected to drying using a GEA Niro Mobile Minor spray-dryer (GEA Process Engineering, Skanderborg, Denmark) equipped with a pneumatic two-fluid nozzle atomizer (1 mm orifice diameter). The inlet and outlet temperatures during drying were 140 °C and 70 °C, respectively, and the pressure of atomization was set to 1.2 bar [50,51]**.**

#### 4.1.3. Chontaduro Gel Ink Preparation With and Without *Lactiplantibacillus plantarum*

Two types of ink were formulated: a chontaduro gel ink control without *Ltp. plantarum* (ChC) and an ink with *Ltp. plantarum* strain (ChLp). The ChC ink consisted of a mixture of 30% (*w*/*w*) freeze-dried chontaduro pulp, 15% (*w*/*w*) whey protein (Colanta, Colombia), and 54.75% (*w*/*w*) water. To this mixture, 0.25% *w*/*w* xanthan gum (Sosa Ingredients S.L., Barcelona, Spain) was added. The water was heated to 30 °C in a heater with a magnetic stirrer (VWR VMS-C7, Munich, Germany), and xanthan gum was added, stirring until it was completely dissolved. Then, it was mixed with chontaduro and whey protein. Before printing, the mixture was tempered to 25 °C. The ChLp ink was reformulated with 27% (*w*/*w*) chontaduro, 13% (*w*/*w*) whey protein, 50% (*w*/*w*) water, and 10% (*w*/*w*) *Ltp. plantarum* carrier.

### 4.2. Characterization of Printing Inks

#### 4.2.1. Rheological Characterization

The rheological behaviour of chontaduro ink was characterized using a HAAKE RheoStress 1 rheometer (Thermo Fisher Scientific, Karlsruhe, Germany). The equipment was configured with a 25 mm diameter rough parallel-plate geometry and a 1.0 mm gap to prevent wall slip. Data acquisition and analysis were performed using RheoWin software V 4.95.0003 (Thermo Fisher Scientific, Karlsruhe, Germany). The linear viscoelastic region (LVR) was determined at a constant frequency of 1 Hz, with the oscillation strain increased logarithmically from 0.1% to 100%. Based on the LVR results, a frequency sweep was performed from 0.1 to 100 Hz at a constant shear stress of 1 Pa. From this frequency sweep, the storage modulus (G′), the loss modulus (G″), the complex modulus (G* in Pa), the complex modulus (G*), and the complex viscosity (η*) were obtained.

#### 4.2.2. Fourier Transform Infrared Spectroscopy (ATR-FTIR)

ATR-FTIR spectroscopy analysis was performed on the samples before printing. The infrared spectra were obtained with a Cary 630 FTIR spectrophotometer (Agilent, Santa Clara, CA, USA) equipped with an ATR (Attenuated Total Reflectance) sampling station. Readings were taken in the wavelength range of 4000–1100 cm^−1^, with a resolution of 16 cm^−1^ and 108 scans of both the background and sample signal. The average spectrum of three subsamples was considered representative of each sample. Spectra were exported to CSV format, and baseline correction of the infrared spectra was performed to compensate for baseline offsets between samples. Multiplicative scattering correction (MSC) was used to counteract light-scattering effects and changes in path length. This processing was performed using the R program (version 4.2.3–2023, R statistics, St. Louis, MO, USA) with the ChemoSpec R-function [52].

### 4.3. Extrusion 3D Food Printer

**Figure 5:**
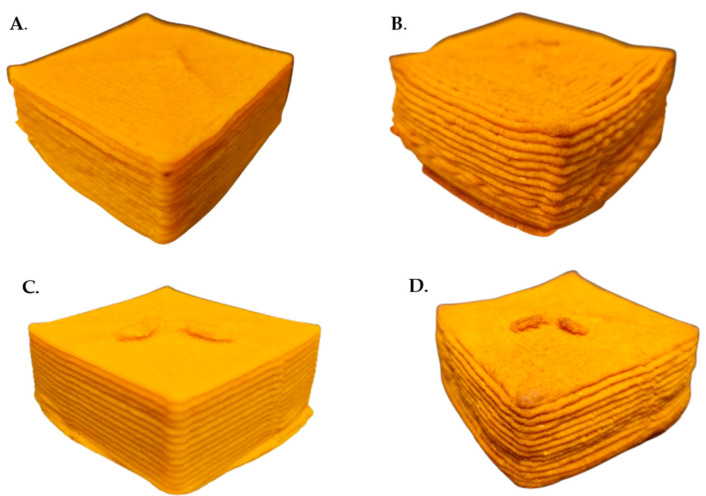
Printed samples. ChC extruded dough (**A**), ChC baked dough (**B**), ChLp extruded dough (**C**), and ChLp baked dough (**D**).

A Foodini printer (Natural Machines, Barcelona, Spain) was used to print the chontaduro gel ink with and without *Lactiplantibacillus plantarum* (Figure 5). To evaluate the printability of the gel, a cube (height = 2.5 cm, width = 5 cm, length = 5 cm) was built in Foodini Creator, and the following printing conditions were set: nozzle diameter 1.5 mm, printing speed 3500 mm/min, ingredient flow 1.7, line thickness 1.4 mm, inter-layer distance 1.4 mm, initial gradient flow 6 mm and ink temperature 25 °C. Each ink was printed in triplicate with separate capsules.

For the sensory test, round shapes (height = 1 cm, radius = 2.5 cm) and square shapes (height = 1 cm, width = 2.5 cm, length = 2.5 cm) were printed from the same dough under the same printing conditions.

### 4.4. Printability Assessment

Images of the top and side views of each freshly printed and freshly baked rectangle were captured with a quad camera at 50 MP on the Galaxy S22+ 5G cell phone (Samsung Electronics Co., Ltd., Suwon, Republic of Korea). To determine shape parameters, these images were processed in ImageJ 1.54g (NIH, Washington, DC, USA). After calibration, five measurements of width, length, and height were taken for each image. The width and height of the rectangle’s base were measured in the front view images, and the length was measured in the upper view images for each sample. Three replicates were performed for each sample. The measured dimensions of the printed rectangles were then compared with the theoretical dimensions, and percentage variation for each parameter was calculated using Equation (1):

(1) $$\%change=\left(\;\frac{A1}{A2}*100\right)-100$$

A1 is the measured geometric characteristic of the printed protein rectangle (width, length, and height) in millimetres, and A2 is the programmed geometric characteristic (width, length, and height) in millimetres. In the same way, the dimensional changes of the baked rectangles relative to the freshly printed rectangles were calculated. In both cases, positive values indicate expansion of the figure, whereas negative values indicate shrinkage.

### 4.5. Water Activity (a_w_)

Water activity was measured in the samples before and after printing and in the baked samples using an electronic water activity meter (LabMaster-a_w_ neo, Novasina AG, Zurich, Switzerland), with the method adapted from AOAC 978.18.

### 4.6. Colour

The colour was determined for the printed and baked samples. Surface colour was evaluated using a CR-400 colourimeter (Konica Minolta Sensing Americas Inc., Ramsey, NJ, USA), and the results were expressed in the CIELab colour space. The parameters measured included L* (lightness, ranging from 0 = black to 100 = white), a* (chromaticity on the green [−] to red [+] axis), and b* (chromaticity on the blue [−] to yellow [+] axis). From these primary coordinates, chroma (C*), representing colour saturation or intensity, and hue angle (h*), indicating the type of colour perceived, were calculated.

In addition, the total colour difference (Δ*E*) was determined to quantify the overall colour change between samples. Δ*E* was calculated using the standard Euclidean distance formula in the CIELab space (2).

(2) $$Total\;color\;difference\;\left({∆E}^{*}\right)=\sqrt{∆L^{*2\;}+∆a^{*2\;}+{∆b}^{*2}}$$

According to [53], colour differences with a ΔE* value less than 1 are undetectable by the human eye. However, smaller colour differences, ranging from 1 to 3 ΔE*, may be discernible depending on the hue. An ΔE* > 3 would be discernible to the human eye.

### 4.7. Texture Profile Analysis (TPA)

The texture properties were measured at room temperature with a TX-700 Texture Analyzer (Lamy Rheology, Champagne au Mont d’Or, France). TPA was performed to measure the hardness, cohesiveness, springiness, and chewiness of the product. Cubes of ChLp extruded dough were axially compressed in two consecutive cycles at a test speed of 2 mms^−1^ at a starting distance of 8 mm with a 25 mm diameter flat plunger.

### 4.8. Survival Assessment of Lactiplantibacillus plantarum Doughs Under Simulated Gastrointestinal Digestion

The survival assessment of *Ltp. plantarum* through the gastrointestinal tract was evaluated using a standardized static in vitro digestion model (INFOGEST protocol). The method was adapted from Minekus et al. [54], with the intestinal electrolyte solution modified as per Mat et al. [53]. Briefly, a 5 g baked chontaduro sample was subjected to sequential oral (2 min with α-amylase), gastric (2 h, pH 3, with pepsin), and intestinal (2 h, pH 7, with pancreatin and bile) phases at 37 °C under constant agitation. The viability of *Lactiplantibacillus plantarum* was assessed after each digestive phase.

### 4.9. Sensorial Analysis

Chontaduro extruded dough samples were baked for 35 min at 140 °C under zero humidity conditions (Rational iCombi Pro, Germany). The study employed a 2 × 2 factorial design, evaluating chontaduro doughs with two shapes (round—RS, square—SS) and two formulations (control (C-) and with *Ltp. plantarum* (CP-)). This resulted in four treatments: CRS (Control Round Shape), CSS (Control Square Shape), CPRS (Chontaduro *Ltp. plantarum* Round Shape), and CPSS (Chontaduro *Ltp. plantarum* Square Shape).

A semi-trained panel (*n* = 28) provided their verbal consent prior to the evaluation. The panellists were informed about the test methodology and the product being evaluated. The panellist used a 7-point scale that was presented in Google forms to evaluate the 13 predefined descriptions (colour: yellow; odour: fruity, fatty, acid, and bitter; taste: fruity, fatty, acid, bitter, and astringent; texture: hard, fibrous, and adhesive).

Assessments were conducted in individual booths under controlled conditions (ISO 8589, 2007). The samples were presented randomly, each with a 3-digit code, on white plates. Mineral water was used to cleanse the palate. The results led to a descriptive profile. This study was approved by the Institutional Ethics Committee of the Universitat Politècnica de València (ref. P06_22-06-2022).

### 4.10. Statistical Analysis

All treatments were performed in triplicate, and the results were reported as the mean ± standard deviation (SD). The rheological curves of G′, G″, G*, and η* were represented as means (*n* = 3) accompanied by their 95% confidence intervals (95% CIs), calculated from the Student’s t-distribution for each combination of treatment and frequency.

Colour, water activity, and textural parameters were evaluated using one-way ANOVA with Statgraphics Centurion XVII (version 19.1.2.0; Statgraphics Technologies, The Plains, VA, USA) at a 95% confidence level (*p* < 0.05). When significant effects were detected, Tukey’s test was applied for mean separation. The survival of *Ltp. plantarum* was analysed by two-way ANOVA in GraphPad Prism Version 10.3.1, followed by Tukey’s post hoc test (*p* < 0.05). Sensory data were analysed using two-way ANOVA with Šídák’s multiple-comparison correction (*p* < 0.05).

## Figures and Tables

**Table 1:** Water activity of ChC and ChLp (extruded dough and baked dough with and without *Lactiplantibacillus plantarum*) at different stages of processing.

Process Stage	ChC	ChLp
Gel	0.976 ± 0.006 ^aX^	0.952 ± 0.001 ^aZ^
Extruded dough	0.962 ± 0.005 ^aX^	0.953 ± 0.003 ^aX^
Baked dough	0.878 ± 0.001 ^cX^	0.841 ± 0.005 ^bZ^

The different lowercase superscript letters (a, b, c) in the same column indicate differences in different states of the food ink under the same treatment, and different uppercase letters (X, Z) in the same row indicate significant differences between treatments (*p* < 0.05) according to Tukey’s HSD test (control chontaduro gel: ChC; chontaduro gel with *Lactiplantibacillus plantarum*: ChLp).

**Table 2:** Colour parameters of chontaduro-based products and effect of *Lactiplantibacillus plantarum* addition and processing.

Process Stage		L*	a*	b*	C*	*h*°
ChLp	ChC	57.1 ± 1.0 ^b^	19.1 ± 0.3 ^c^	60.5 ± 0.9 ^b^	63.5 ± 1.0 ^b^	252.5 ± 0.1 ^ab^
	ChLp	58.7 ± 0.6 ^a^	19.6 ± 0.2 ^bc^	64.3 ± 0.5 ^a^	67.2 ± 0.5 ^a^	253.1 ± 0.1 ^a^
Extruded Dough	ChC	57.2 ± 0.5 ^b^	19.9 ± 0.2 ^b^	60.6 ± 1.1 ^b^	63.7 ± 1.1 ^b^	251.8 ± 0.1 ^b^
	ChLp	59.5 ± 0.3 ^a^	19.3 ± 0.2 ^bc^	62.5 ± 0.9 ^a^	65.4 ± 0.9 ^ab^	252.8 ± 0.4 ^a^
Baked Dough	ChC	51.7 ± 0.5 ^c^	22.3 ± 0.4 ^a^	56.3 ± 0.5 ^c^	60.6 ± 0.4 ^c^	248.4 ± 0.5 ^c^
	ChLp	44.5 ± 0.6 ^d^	19.7 ± 0.5 ^bc^	43.1 ± 2.1 ^d^	47.4 ± 2.0 ^d^	245.4 ± 0.9 ^d^

Values are expressed as mean ± standard deviation (*n* = 3). For each parameter (column), different lowercase superscript letters (a–d) indicate significant differences between all formulation and processing condition combinations based on Tukey’s test (*p* < 0.05). L*: lightness; a*: red/green coordinate; b*: yellow/blue coordinate; C*: chroma; *h*°: hue angle (control chontaduro gel: ChC; chontaduro gel with *Lactiplantibacillus plantarum*: ChLp).

**Table 3:** Textural profile analysis of chontaduro doughs with and without microorganisms at different processing stages.

Process Stage		Hardness (N)	Cohesiveness	Springiness (mm)	Gumminess (N X mm)	Chewiness (N X mm)
Extruded Dough	ChC	13.8 ± 1.4 ^c^	0.2 ± 0.1 ^a^	0.6 ± 0.4 ^a^	2.7 ± 0.9 ^c^	0.7 ± 0.3 ^b^
	ChLp	17.0 ± 0.3 ^c^	0.10 ± 0.01 ^a^	0.5 ±0.2 ^a^	1.80 ± 0.04 ^c^	0.8 ± 0.3 ^b^
Baked Dough	ChC	80.8 ± 1.4 ^b^	0.13 ± 0.03 ^a^	0.4 ±0.1 ^a^	9.0 ± 0.1 ^b^	3.0 ± 0.5 ^b^
	ChLp	119.5 ± 17.7 ^a^	0.16 ± 0.02 ^a^	0.6 ± 0.2 ^a^	20.4 ± 4.6 ^a^	13.8 ± 3.1 ^a^

Values are expressed as mean ± standard deviation (*n* = 3). For each parameter (column), different lowercase superscript letters (a–c) indicate significant differences between chontaduro doughs with and without microorganisms for textural parameter based on Tukey’s HSD test (*p* < 0.05) (control chontaduro gel: ChC; chontaduro gel with *Lactiplantibacillus plantarum*: ChLp).

**Table 4:** Survival assessment of *Lactiplantibacillus plantarum* in chontaduro gel, extruded dough, and baked dough under simulated gastrointestinal conditions.

Process Stage	Cell Viability [Log (CFU/g)]		
	Before Digestion	Oral Phase	Gastric Phase
ChLp	7.0 ± 0.2 ^aX^	6.8 ± 0.3 ^aX^	5.6 ± 0.8 ^aZ^
ChLp Extruded Dough	7.1 ± 0.1 ^aX^	6.9 ± 0.1 ^aX^	4.7 ± 0.2 ^aZ^
ChLp Baked Dough	0.0 ^b^	0.0 ^b^	0.0 ^b^

The different lowercase superscript letters (a, b) in the same column indicate differences in different states of the food ink under the same treatment, and different uppercase letters (X, Z) in the same row indicate significant differences between treatments (*p* < 0.05) according to Tukey’s HSD test.

## Data Availability

The data supporting the findings of this study are available from the corresponding author upon reasonable request. The data are not publicly available because they are part of an ongoing research project.

## Abbreviations

The following abbreviations are used in this manuscript:

ChC	Control chontaduro gel
ChLp	Chontaduro gel with Lactiplantibacillus plantarum
CRS	Control Round Shape
CSS	Control Square Shape
CPRS	Chontaduro-based product with Lactiplantibacillus plantarum with a round shape
CPSS	Chontaduro-based product with Lactiplantibacillus plantarum with a square shape
FTIR	Fourier Transform Infrared Spectroscopy
